# Percutaneous dilatational tracheostomy: complications and safety without the use of bronchoscopic guidance

**DOI:** 10.1186/cc14294

**Published:** 2015-03-16

**Authors:** R Johnsen

**Affiliations:** 1 Sygehus Lillebælt, Vejle, Denmark

## Introduction

Since the Introduction and development of percutaneous dilatational tracheostomy (PDT), this procedure is accepted and incorporated in ICUs worldwide. In spite of obvious benefits for the patients, who obtain more comfort and mobility and less use of sedatives, the procedure also implies the risk of several complications, some of which may be lethal. Severe complications include hemorrhage, displacement and pneumothorax. Different

## Methods

of PDT are described in the literature, each with disadvantages and benefits. The aim of this study was to analyze complications due to PDTs performed without the use of bronchoscopic assistance.

## Methods

The study was conducted in a Danish eight-bed, non-university ICU. Since 2007, all patients admitted to the ICU have been registered on an electronic patient record system, in which daily vital values, diagnoses, procedures and healthcare providers' notes are entered. When searching for 'percutaneous dilatation tracheostomy' in the electronic system, we found all patients who had undergone this specific procedure. Afterwards we analyzed each of these patients' hospital records, looking for any periprocedure or postprocedure complications noted within 7 days. In addition we registered patients' age, sex, BMI, SOFA score, methods used in procedures and experience of operators.

## Results

A total of 136 patients admitted to the UCI had undergone a PDT between 2007 and 2014. Of these, two were excluded due to the PDT being performed in another hospital before admission to our ICU. All 134 PDTs were performed with the Ciaglia Blue Rhino Method. No PDTs were performed with bronchoscopic guidance. In 12 cases some kind of complication due to the PDT was registered: six cases with need of surgical hemostasis, three cases of bleeding with need of transfusion of blood products, one case of PDT displacement, one case of ventilation-related problems during procedure and, finally, one case of tracheal cartilage fracture. There were no incidents of pneumothorax. No PDTs had a lethal outcome due to the procedure itself. The total complication rate was 9.0%. Of the 12 cases, four (33%) complications occurred during the procedure, the rest (66%) occurred after the procedure. The overall periprocedure complication rate was 3%.

## Conclusion

In this study, PDTs without the use of bronchoscopic guidance were performed safely with a low rate of complications.

